# Primary dedifferentiated liposarcoma of the femur presenting with malignant fibrous histiocytoma: A case report and review of the literature

**DOI:** 10.3892/ol.2014.2150

**Published:** 2014-05-16

**Authors:** SHUAI ZHANG, XU-QUAN WANG

**Affiliations:** Department of Orthopedic Surgery, Southwest Hospital, Third Military Medical University, Chongqing 400038, P.R. China

**Keywords:** liposarcoma, femur, pathology

## Abstract

In the current report a case of a 26-year-old male with a primary dedifferentiated liposarcoma of the femur accompanied by malignant fibrous histiocytoma is presented. The patient complained of a dull intermittent pain, for three months, along the anterolateral aspect of the right knee and was referred to Southwest Hospital (Chongqing, China) in May 2013. All of the radiographic findings, including radiography, magnetic resonance image and emission computed tomography (CT) supported the diagnosis of a primary malignant bone tumor. CT-guided biopsy results demonstrated blood clots and a small quantity of heterogeneous cells. Thus, a limb-salvage procedure, involving a wide resection and total knee endoprosthesis replacement, was performed in May 2012. The final pathological diagnosis was of a primary dedifferentiated liposarcoma of the femur and the dedifferentiated tissue was identified as malignant fibrous histiocytoma. On review of the literature, it was identified that primary intraosseous liposarcoma are a rare and malignant tumor of the skeletal system, with only a small number of cases reported in the English literature since 1980. To the best of our knowledge, a case of dedifferentiated liposarcoma has not previously been reported.

## Introduction

Primary intraosseous liposarcoma is a rare and malignant type of skeletal system tumor; the first case was reported in 1931 by Stewart (1). Primary intraosseous liposarcoma originate from the intraosseous adipose tissue. Since 1980, only a small number of cases of primary liposarcoma involving a long bone have been described in the English literature. Recently, a patient exhibiting dedifferentiated liposarcoma presented at the Southwest Hospital (Chongqing, China), and all of the radiographic, clinical and pathologic evidence indicated a primary bone origin. The purpose of the present report is to increase awareness of this uncommon type of malignant tumor through the description of the clinical, surgical, radiographic and pathologic findings observed in this particular patient. Patient provided written informed consent.

## Case report

**Figure 1:**
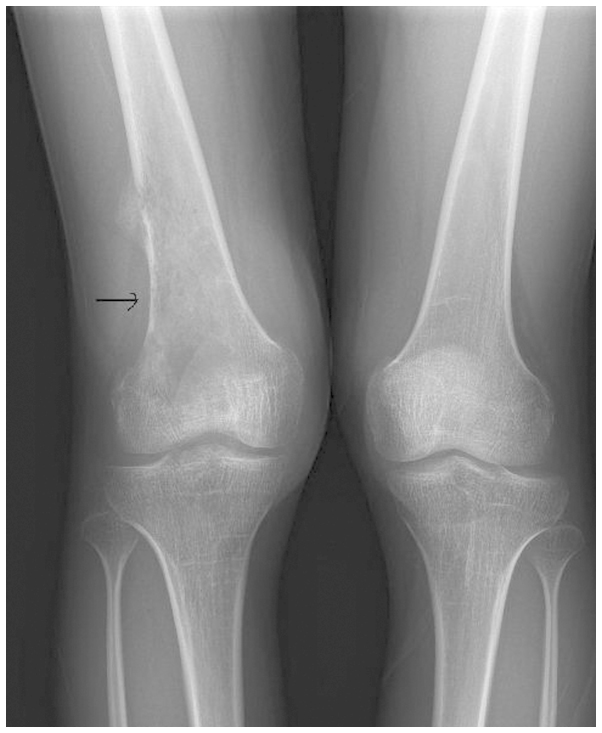
Anteroposterior radiograph of the distal right femur showing a destructive lesion (arrow), with lateral cortical discontinuity and soft-tissue extension.

**Figure 2:**
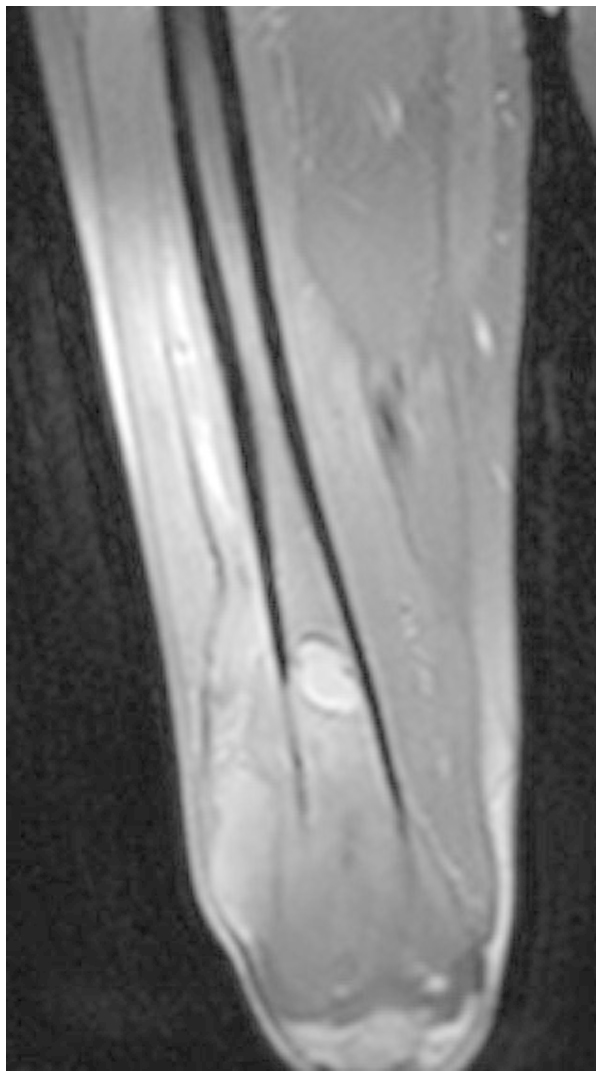
Magnetic resonance image demonstrating the osteolytic lesion in the femur and diffuse erosion of the cortex, with involvement of the surrounding soft-tissues.

**Figure 3:**
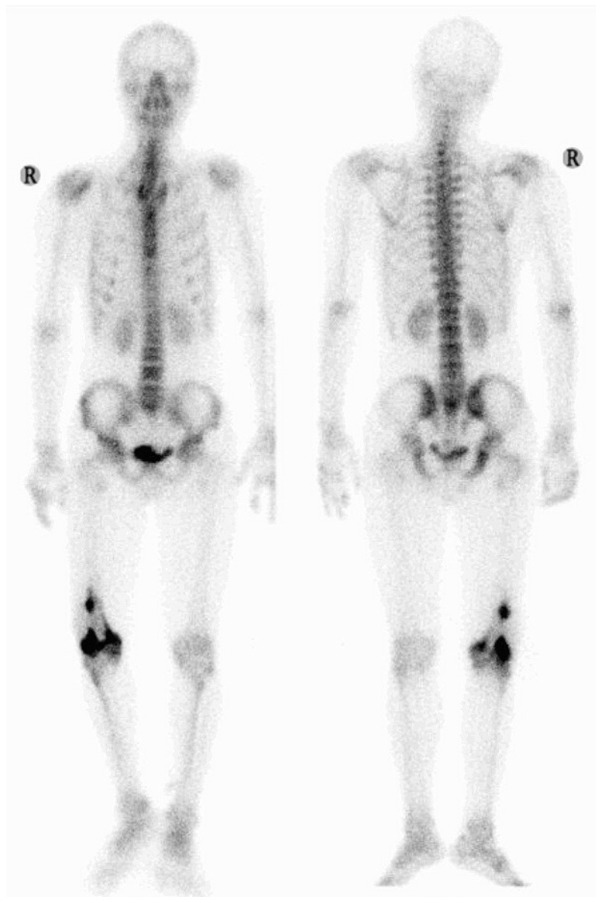
Emission computed tomography bone scan showing an abnormal, isolated concentration of the radioactive agent in the distal right femur.

**Figure 4:**
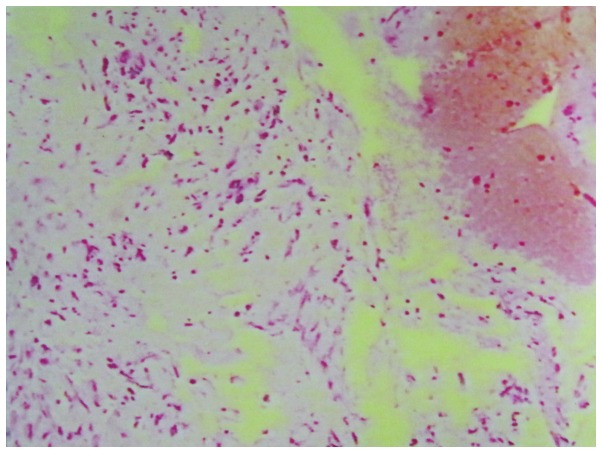
Computed tomography-guided biopsy results showing a small number of heterogeneous cells (hematoxylin and eosin stain; magnification, ×200).

A 26-year-old male presented at the Department of Orthopedic Surgery, Southwest Hospital, complaining of a dull intermittent pain, for three months, along the anterolateral aspect of the right knee. The pain was relieved by taking Celebrex^®^, however, it was exacerbated by activity. The patient had identified a slow-growing mass on the outside of the right lower thigh and, a month prior to admission, the patient had noted a moderate sensation of warmth in this region. There was no history of accident, injury, fever, weakness or weight-loss. The patient visited another hospital in April 2012 and radiographs demonstrated a tumorous lesion in the right distal femur. The patient was referred to the Southwest Hospital in May 2012 with an unremarkable medical history. However, the physical examination was notable due to swelling, a decreased range of motion of the right knee and a palpable mass (size, ~5×4×3 cm). The laboratory assessments of the patient’s alkaline phosphatase levels were unremarkable. The radiographs revealed an expansile and osteolytic lesion of the right distal femur with a periosteal reaction (Fig. 1). Magnetic resonance imaging demonstrated an expansile, intramedullary, poorly defined neoplasm with a moderately high signal intensity area on T1- and T2-weighted images in the distal part of the femur, with diffuse erosion of the cortex and involvement of the surrounding soft tissue (Fig. 2). The intraosseous central location and uniform cortical destruction indicated that the lesion was not a soft-tissue sarcoma, which are associated with bone invasion. An emission computed tomography (CT) bone scan demonstrated an abnormal isolated concentration of radioactive agent (used for bone imaging) at the femoral site, which revealed that no distant metastases had occurred (Fig. 3). Chest CT did not demonstrate any thoracic abnormality and the CT-guided biopsy results showed blood clots as well as a small quantity of heterogeneous cells (Fig. 4). Therefore, the clinical diagnosis was a malignant bone tumor, with the most likely diagnosis considered to be an osteosarcoma, as a primary bone tumor.

**Figure 5:**
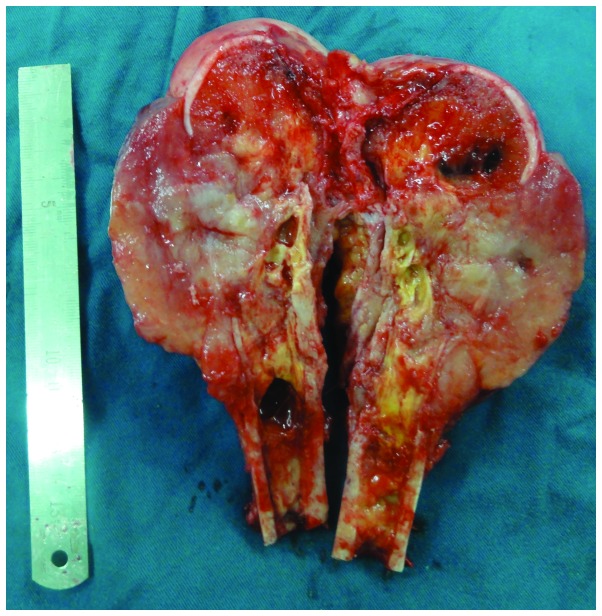
A variety of colors (light yellow, gray and gray/red) with necrosis, were visible on the cut surface of the specimen.

A limb-salvage procedure, involving a wide resection and a total knee endoprosthesis replacement, was performed in May 2012. Examination of the resection specimen showed a soft, light-yellow (or gray), gelatinous tumor measuring 13 cm along the long axis of the femur and 6 cm transversely. The tumor was centered in the distal part of the femur, with involvement of the surrounding soft tissues. A central hemorrhagic and necrotic area contained serosanguineous fluid, with an abundant local blood supply (Fig. 5). No intra-articular extension was observed and there was no indication of regional metastasis on dissection of the popliteal fossa lymph nodes. The patient’s postoperative course was uneventful.

**Figure 6:**
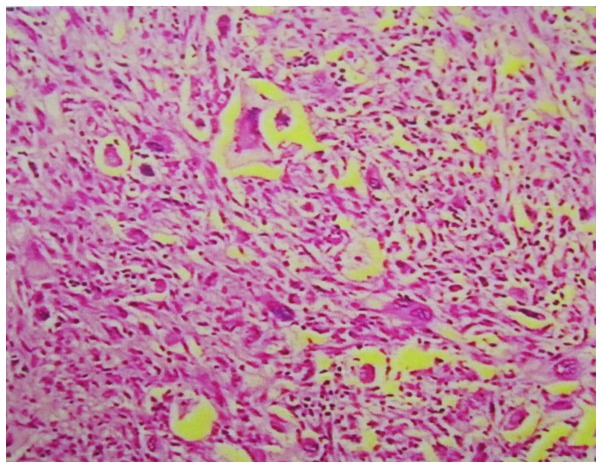
Photomicrograph of the tumor, showing fatty tissue with varying degrees of differentiation and many giant tumor cells. Ovoid and polygonal tumor cells were of diffuse distribution, with large and condensed nuclei, obvious atypia and mitotic figures (hematoxylin and eosin stain; magnification, ×200).

Microscopically, the tumor was identified as a sarcoma, which demonstrated ovoid and polygonal tumor cells that were of diffuse distribution with obvious atypia and mitotic figures. In addition, a combination of numerous giant tumor cells and fatty tissue with different degrees of differentiation was observed (Fig. 6). Immunohistochemistry showed positive staining for S-100 protein and cluster of differentiation (CD)68, and negative staining for smooth muscle actin, CD34 and vimentin. The final pathological diagnosis was a primary dedifferentiated liposarcoma of the femur, and the dedifferentiated tissue was identified as malignant fibrous histiocytoma.

No other types of therapy, including chemotherapy and radiation, were selected prior to and following surgery. The patient was followed up for 12 months after surgery, and no recurrence and metastasis was detected. In addition, radiography indicated that the artificial joint was in good condition.

## Discussion

Liposarcoma is a common type of sarcoma that affects the soft tissues, however, primary intraosseous liposarcomas are particularly rare (2) with only 10 cases described in previous reports since 1980 (Table I) (3–12). To the best of our knowledge, no cases of primary dedifferentiated liposarcoma of the bone have been reported.

Table I indicates that the long bone is most commonly affected by liposarcoma, particularly in the humerus and there has only been one case where the lesion occurred in the femur. As with other malignant tumors of the bone, patients present with a history of pain, swelling, decreased range of motion, and an expansile and osteolytic lesion as demonstrated by radiographs. Clinically, it is possible to mistake a primary intraosseous liposarcoma for an osteosarcoma, Ewing’s sarcoma, plasmacytoma or a lymphoma. Primary intraosseous liposarcoma can only definitively be diagnosed by pathological methods. The initial observations of the biological behavior of the lesion, the imageology data and past experience indicated that the present case was exhibiting an osteosarcoma. However, the final pathological diagnosis following surgery was a dedifferentiated liposarcoma.

Due to the limited quantity of previous cases and the various treatment modalities that were used, there is no definitive standard treatment for primary intraosseous liposarcoma and it is difficult to formulate an effective treatment protocol. Surgical resection remains the primary treatment method. To reduce recurrence, Mouret (13) proposed that during complete resection of liposarcoma a margin around the lesion of ≥2 cm is required, as satellite nodules are occasionally present within this margin. However, complete resection may be difficult in certain regions, for example the head and neck, due to the presence of critical organs. Chemotherapy is another treatment modality selected by certain clinicians rather than surgery. Although the first case in 1982 reported the use of chemotherapy as the treatment strategy (5), a total of five cases have been reported (5,6,8,10,12), where combined-treatment chemotherapy was adopted; the survival times for these patients were 10, 16, 70, 7 and 3 months, respectively. In 1999, Rabah *et al* (8) reported the case of a patient with a liposarcoma of the humerus. The patient was systematically administered with adjuvant chemotherapy, which comprised of four cycles of cisplatin, doxorubicin and ifosfamide prior to surgery, and continued for five further cycles following surgery. The patient survival time of 70 months was the longest. Thus, chemotherapy may be an additional optional adjuvant treatment.

Liposarcoma is commonly classified into five pathological categories, as follows: Well-differentiated; dedifferentiated; round cell; myxoid; and pleomorphic (14). The histological classification is important for treating liposarcoma, as the clinical features and prognosis are dependent on it. Enzinger and Weiss (14) reported that the five-year survival rate of patients with well-differentiated liposarcoma is ~90%, while that of patients with the myxoid type is ~80%, pleomorphic type is ~20% and the round cell type is <20%. There is no five-year survival rate for the dedifferentiated type, as few cases have been reported.

In conclusion, based on Table I, it is apparent that the majority of patients with primary intraosseous liposarcoma succumb to this metastatic disease, with the common metastatic site being the lungs, indicating that this type of tumor is associated with a poor prognosis. Therefore, careful observation during follow-up is recommended to ensure the early detection of recurrence and distant metastasis. The patient in the present study returned for follow-up one year following surgery, and no recurrence and metastasis was detected. To the best of our knowledge, this is the first report to present a case of dedifferentiated primary liposarcoma of the bone.

## Figures and Tables

**Table I:** Primary liposarcoma of various bones as reported in the English literature since 1980.

First author (Ref.)	Year	Age (years)/Gender	Site	Subtype	Treatment	Metastasis	Outcome (months)
Schneider (3)	1980	69/M	Fibula	Unknown	Amputation	(-)	Unknown (24)
Bolen (4)	1981	39/M	Humerus	Pleomorphic	Humeroscapular disarticulation	(-)	Unknown
Addison (5)	1982	19/M	Humerus	Pleomorphic	Amputation, radiation, chemotherapy	Lung	Succumbed (10)
Torok (6)	1983	34/M	Femur	Pleomorphic	Wide resection, radiation, chemotherapy	Lung	Succumbed (16)
Kenan (7)	1991	57/M	Scapula	Myxoid	Curettage	Supra-clavicular	Alive (36)
Rabah (8)	1999	16/F	Humerus	Pleomorphic	Chemotherapy, limb salvage procedure	Liver	Succumbed (70)
Hamlat (9)	2005	45/F	Thoracic	Pleomorphic	Radiation, vertebrae decompressive laminectomy resection, endoporosthesis replacement	Lung, rib	Alive (6)
Torigoe (10)	2006	38/F	Humerus	Pleomorphic	Chemotherapy	Liver	Succumbed (7)
Seo (11)	2007	69/M	Temporal bone	Well- differentiated	Palliative resection	(-)	Alive (24)
Lmejjati (12)	2008	45/M	Lumbar vertebrae	Pleomorphic	Decompressive laminectomy, novel adjuvant chemotherapy, limb salvage	Unknown	Succumbed (3)

M, male; F, female; -, no metastasis.
